# Cutaneous presentation of cryptococcal infection with subclinical central nervous system involvement secondary to fingolimod therapy

**DOI:** 10.1177/20552173231197132

**Published:** 2023-09-03

**Authors:** Shin Yee Chey, Niamh-Anna O’Sullivan, Trevor Beer, Wai K Leong, Allan G Kermode

**Affiliations:** Department of Neurology, 5728 Sir Charles Gairdner Hospital, Perth, Western Australia, Australia; Perron Institute for Neurological and Translational Sciences, QE II Medical Centre, Perth, Australia; 7800 University of New South Wales, Sydney, New South Wales, Australia; 547049 Clinipath Pathology, Osborne Park, Australia; Department of Neurology, 6508 Royal Perth Hospital, Perth, Western Australia, Australia; Perron Institute for Neurological and Translational Sciences, QE II Medical Centre, Perth, Australia

**Keywords:** Fingolimod, cutaneous cryptococcal infection, cryptococcal meningitis, central nervous system cryptococcal infection, multiple sclerosis

## Abstract

Fingolimod is a multiple sclerosis disease-modifying therapy which sequestrates lymphocytes in the lymph nodes, thereby reducing peripheral blood lymphocytes. Cryptococcal infection is an important adverse effect which should be recognised. We report a case of cutaneous and central nervous system infection who presented with isolated cutaneous symptoms in the absence of neurological or systemic manifestations.

## Case report

**Figure 1.**
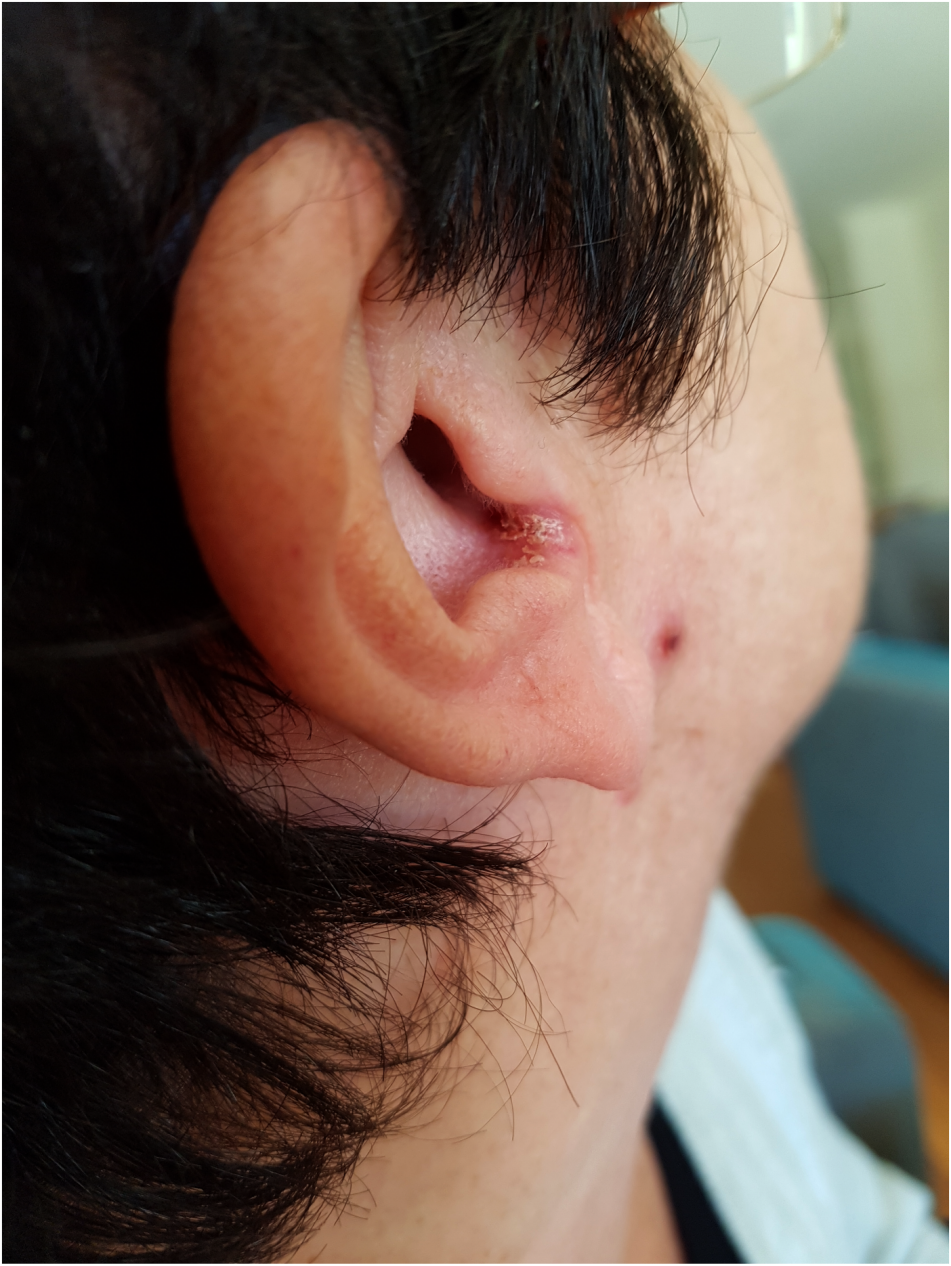
Showing a discrete hyperkeratotic erythematous plaque at the right intertragal notch.

**Figure 2.**
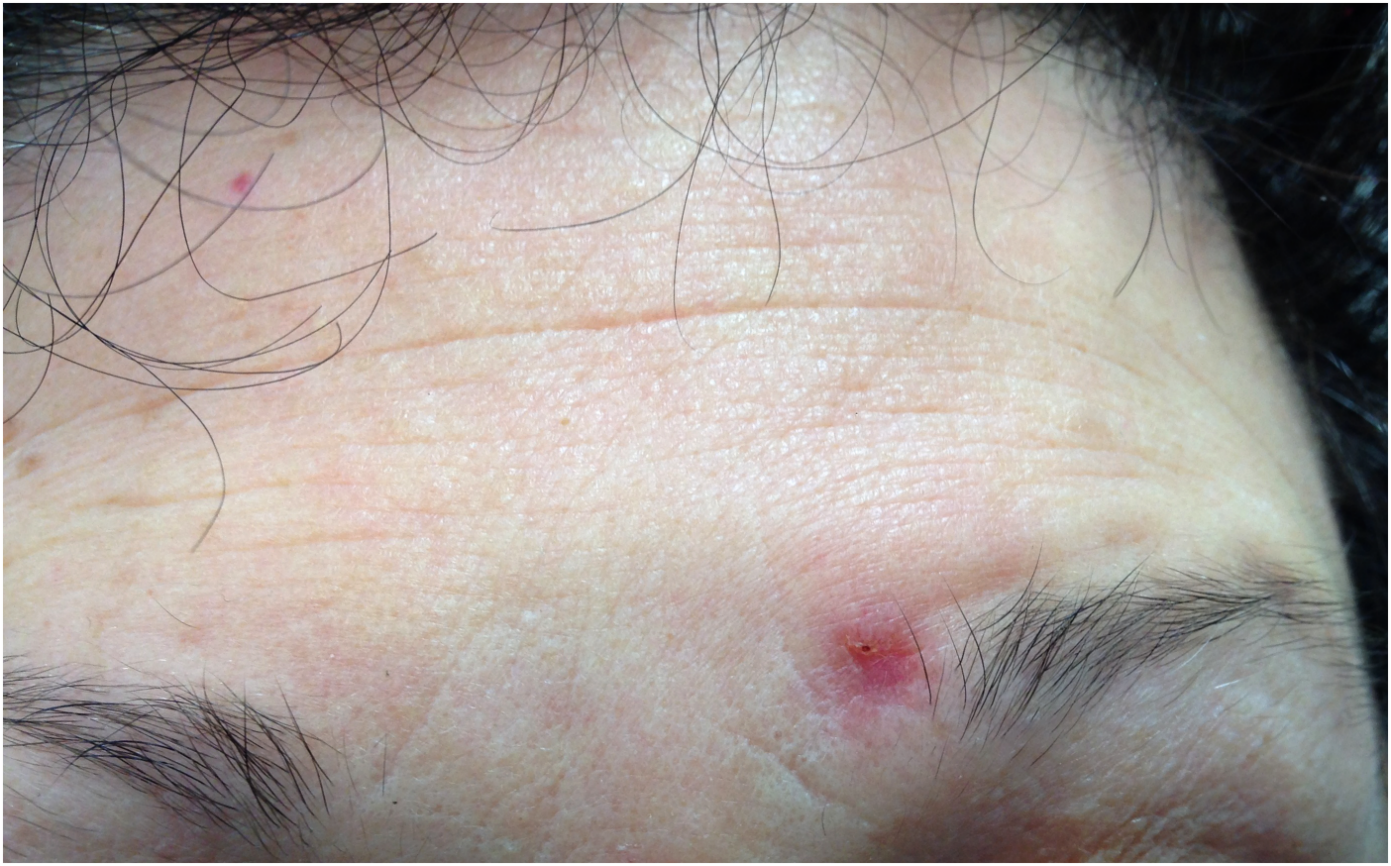
Showing an erythematous, smooth, subtly atrophic plaque with a solitary punctate haemorrhagic erosion at the left medial eyebrow.

**Figure 3.**
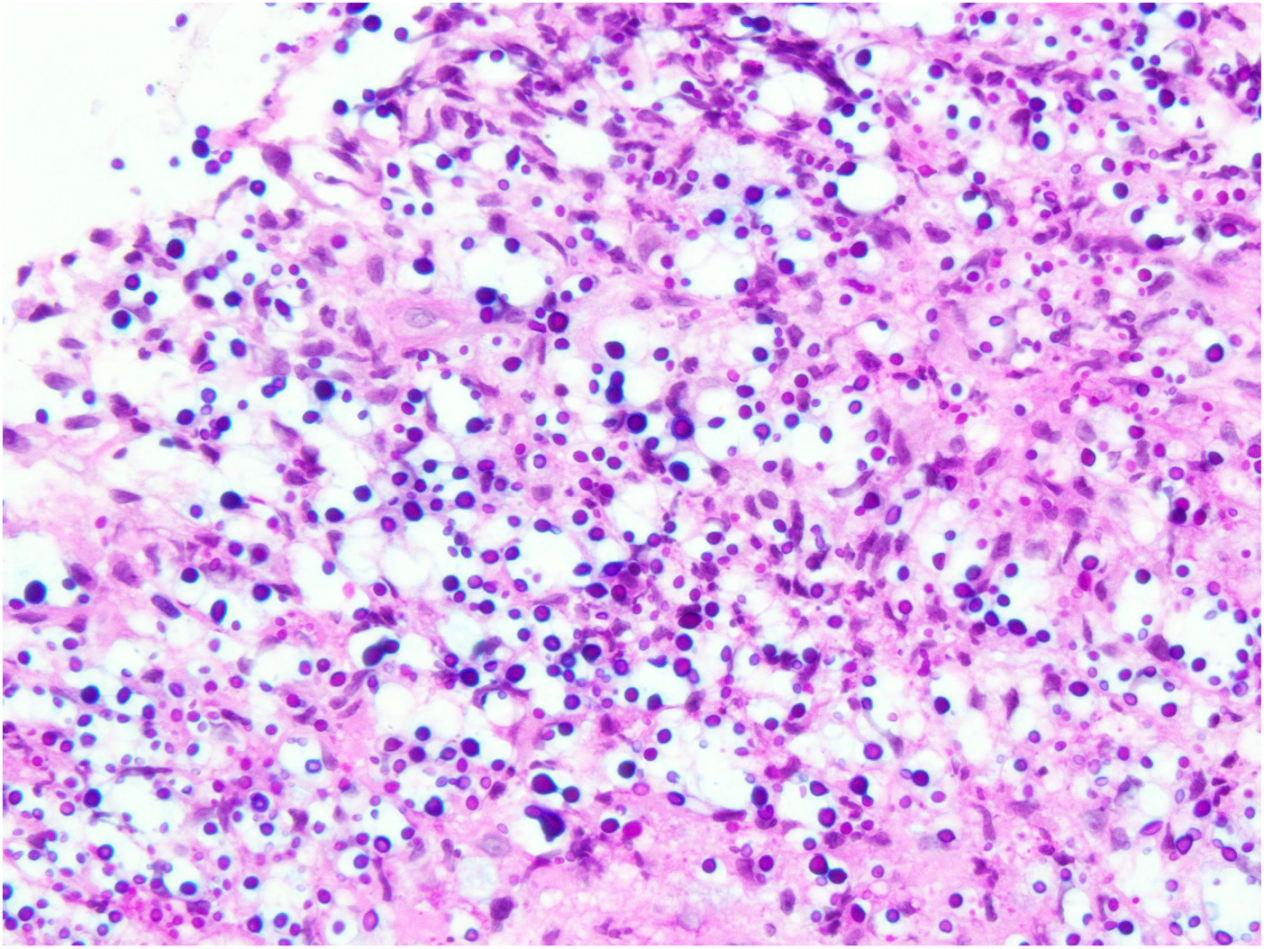
Mucicarmine histochemistry staining showing numerous multinucleated giant cells containing carmininophilic yeasts with clear halo around each yeast.

A 56-year-old female with MS who was treated with fingolimod developed a non-healing rash over the left brow and a crusty lesion over the right ear 45 months following the initiation of the treatment. She denied other neurological or systemic features. The patient's MS remained relapse-free but she developed significant lymphopenia with fingolimod, with a nadir lymphocyte count of 0.2 × 10^9^/L. Her significant past medical history included breast cancer (invasive ductal carcinoma) for which she underwent a left mastectomy 3 years prior, and completed chemotherapy (fluorouracil, epirubicin, cyclophosphamide and docetaxel), followed by maintenance tamoxifen therapy. Post-chemotherapy and prior to initiation of fingolimod, her lymphocyte subsets and immunoglobulin levels were normal. On examination, the patient had a solitary hyperkeratotic erythematous plaque at the right intertragal notch (Figure 1), and an erythematous, smooth, subtly atrophic plaque with a solitary punctate haemorrhagic erosion at the left medial eyebrow (Figure 2). A skin biopsy was performed, the results of which were consistent with cryptococcal infection (Figure 3). The patient's MRI Brain did not show leptomeningeal enhancement. A lumbar puncture was performed, showing a normal opening pressure and an elevated cerebrospinal fluid (CSF) protein of 0.87 g/L, CSF glucose 2.9. There was significant CSF pleocytosis with 160 × 10^6^ leucocytes. Indian Ink study showed encapsulated cells. Both serum and CSF cryptococcal antigens were positive. Cryptococcus neoformans were cultured from CSF. Fingolimod was ceased which was followed by gradual recovery of her lymphopenia. The patient completed a total of 12 months of amphotericin and fluconazole. The patient was started on peginterferon beta-1a 8 months later.

## Discussion

Cryptococcal infection predominantly occurs in patients who are immunocompromised such as patients with HIV or those who receive immunosuppressive therapy. Fingolimod is a sphingosine 1 receptor inhibitor which prevents the egress of lymphocytes from the lymph nodes, thereby reducing the circulating lymphocytes, and this increases the risks of infections. Fingolimod has been shown to be associated with a higher incidence of herpes zoster, lower respiratory tract, and influenza infections.^1^ Cases of cryptococcal infection have also been increasingly reported. This case highlights the importance of recognising cryptococcal infection as a complication of fingolimod therapy.

The actual incidence of cryptococcal infections in patients receiving fingolimod is not known, and there may have been unreported cases. From our literature review, there have been 17 other published cases (Table 1) of cryptococcal infections related to fingolimod use. The spectrum of fingolimod-related cryptococcal infection ranged from isolated asymptomatic pulmonary or cutaneous cryptococcosis to meningitis and disseminated cryptococcal infection. A majority of the cases with cryptococcal meningitis presented with typical symptoms and signs of cryptococcal meningitis such as headache or altered mental status. Patients ranged from 34 to 67 years of age. The mean duration of fingolimod therapy was 56.4 months (24 to 144 months). A majority of the patients did not have other identifiable risk factors for cryptococcal infection. Only a small number of the patients were immunocompromised due to primary immunodeficiency or being on another immunosuppressive agent. Published cases of cryptococcal infection associated with fingolimod had varying degrees of lymphopenia, with lymphocyte count ranging between 0.09 × 10^9^/L to 2.2 × 10^9^/L.

**Table 1.** Published cases of cryptococcal infections related to fingolimod use.

No	Age/gender	Site of infection	Lymphocyte count (×10^9^/L)	Duration of fingolimod (months)	Other risk factors	Reference
1	40/M	CNS	0.4	24	–	^2^
2	62/M	CNS	0.33	36	–	^3^
3	49/F	CNS	0.09	66	IgG2 deficiency	^4^
4	62/F	Cutaneous	0.65	36	–	^5^
5	52/M	Cutaneous and CNS	0.5	42	–	^6^
6	67/F	CNS	2.39	41 (symptom onset after 6–8 weeks after discontinuation of fingolimod)	–	^7^
7	34/M	CNS	NA	60	–	^8^
8	61/F	CNS	0.12	36	–	^9^
9	63/M	Cutaneous	NA	84	–	^10^
10	49/M	Cutaneous	0.35	NA	–	^11^
11	48/F	CNS	0.21	91	On IV Abatacept 1000 mg monthly for RA	^12^
12	46/F	Chest wall abscess and rib osteomyelitis	0.3	144	–	^13^
13	45/M	Pulmonary	0.68	36	–	^14^
14	40/F	CNS	2.2	27	–	^15^
15	41/F	CNS	0.177	72	–	^16^
16	58/M	CNS	0.9	84	–	^17^
17	63/M	Cutaneous and CNS	0.5	24	–	^18^

M: male, F: female, CNS: central nervous system, NA: not available, RA: rheumatoid arthritis.

In our patient, her nadir lymphocyte count was 0.2 × 10^9^/L, and it is plausible that this increases her risk of infection. In our opinion and based on reported cases in the literature, most patients have been on the treatment for a duration longer than 24 months, and a large proportion of the patients came from an older age group.

Interestingly, despite proven CNS involvement, our patient presented with cutaneous instead of neurological or systemic symptoms. Primary cutaneous cryptococcosis related to fingolimod therapy is rare as there had only been three reported cases to our knowledge. This case highlights a rare presentation of cutaneous cryptococcosis in a patient receiving fingolimod. We would also like to emphasise the importance of ruling out CNS infection in patients with cutaneous cryptococcal infection despite an absence of neurological symptoms as cryptococcal meningitis is often associated with a high mortality, and the treatment will include the commencement of amphotericin rather than itraconazole which can be used in primary cutaneous cryptococcal infection. We proceeded with a cranial MRI and a lumbar puncture in this case, and the CSF confirmed cryptococcal neoformans infection.

## Conclusion

Our reports aim to raise awareness of cryptococcal infection as a not-to-be-missed complication of fingolimod. Although a majority of the reported cases present with typical symptoms associated with meningism, we would like to highlight that the spectrum of presentation may vary, and that patients may be relatively asymptomatic despite CNS involvement at least in the early stages. As cryptococcal infection is associated with significant morbidity and mortality if left untreated, the importance of timely diagnosis and early institution of treatment in this group of patients must be emphasised.
